# Analysis of 461 Consecutive Patients' Donor Site Morbidity following Abdominal Tissue-Based Breast Reconstruction without Fascia Reinforcement Graft

**DOI:** 10.1155/2022/7221203

**Published:** 2022-02-15

**Authors:** Seong Ae Kim, Daheui Kim, Deuk Young Oh, Jung Ho Lee

**Affiliations:** Department of Plastic and Reconstructive Surgery, College of Medicine, The Catholic University of Korea, Seoul, Republic of Korea

## Abstract

Despite the popularity of breast reconstruction with abdominal flap, the integrity of the abdominal wall gets compromised after the operation. To decrease donor site morbidity, researchers have developed various inlay or onlay graft materials. However, the indications of use are unclear and dependent on the subjective decision of the surgeons. In this study, we have investigated donor site morbidities in breast reconstruction with free abdominal flap surgery in which graft materials were not used. We reviewed 461 consecutive cases for the preoperative characteristics of patients, intraoperative details, and postoperative donor site complications from May 2013 to March 2019. While 386 patients underwent deep inferior epigastric perforators (83.7%), muscle sparing type 2 transverse rectus abdominis musculocutaneous flaps were performed in 75 patients (16.3%). Bilateral dissection of the pedicle was performed in 162 patients, compared to unilateral dissection in 299 patients. The mean follow-up duration was 22.7 months. The overall complication rate in the donor site was 7.2%. The flap height was significantly associated with the overall complication. While majority of them were delayed wound healing (*n* = 28, 6.1%), there were four cases of hematoma (0.9%). There were two cases of bulging (0.4%), which occurred in patients receiving bipedicle dissection; however, there was no case of hernia. *Conclusions*. Breast reconstruction with an abdominal free flap can be safely performed without fascia reinforcement graft even with bilateral dissection of the pedicle. With complete preservation of fascia and zigzag fascial incision, a low incidence of abdominal bulging can be obtained even with bilateral harvesting of the flap.

## 1. Introduction

Despite the evolution of the surgical technique, which decreases the damage in muscle and fascia, reconstruction with abdominal free flap may result in abdominal bulging and hernia. Notwithstanding the variations, the rates of abdominal flap complications range from 4% to 20% and 0.7% to 5% for free transverse rectus abdominis musculocutaneous (TRAM) flap and deep inferior epigastric perforators (DIEP) flap, respectively [1–3].

Researchers have developed various inlay graft materials, such as polypropylene mesh or a bioprosthetic material (acellular dermal matrix), which decrease the donor site morbidity and strengthen the abdominal fascia [4, 5]. The use of graft reduces the tension at the fascial repair site, facilitates midline position of umbilicus, and avoids an asymmetrical lower abdominal contour [4]. However, they impose an extra financial burden on the patients and can lead to infection or delayed wound healing. Moreover, the indication of use is unclear and dependent on the subjective decision of the surgeons.

We aimed to investigate the donor site morbidity and related factors in breast reconstruction with free abdominal flap surgery. In particular, bilateral dissection cases were included at a high rate, and the effect of these on donor site complications was investigated. All free abdominal flaps were harvested with the complete preservation of rectus fascia. Moreover, the fascia was closed primarily without any inlay or onlay graft materials.

## 2. Patients and Methods

Our retrospective study was approved by the institutional review board. We enrolled 461 consecutive patients with DIEP and TRAM from May 2013 to March 2019. We retrospectively reviewed all known preoperative risk factors for donor site morbidity, such as smoking, diabetes, American Society of Anesthesiologists classification, body mass index (BMI), previous abdominal operation history, and neoadjuvant chemotherapy. In addition, we recorded the intraoperative details, including the height of flap, relative height ratio of flap, flap weight, and bilateral or unilateral dissection of pedicle. Relative height ratio was defined as the flap height divided by the distance between the symphysis pubis and xiphoid process. We categorized the patients as unilateral or bilateral based on the pedicle dissection from one or both sides of the abdominal wall: the ‘bilateral' category included patients with bilateral breast reconstruction and unilateral breast reconstruction with super or turbo charged flap. We identified the postoperative incidence of hernia and bulging. Abdominal bulging was defined as a palpable bulge on physical examination. Nonetheless, there was no fascial defect on computed tomography (CT) scans. We defined abdominal hernia as a bulging with fascial defect as confirmed on CT scans. In addition, the incidence of other complications, such as infection, delayed wound healing, hematoma, and seroma, was also investigated. Delayed wound healing was defined as those necessitating surgical revision or >2 weeks of dressing for complete healing.

## 3. Surgical Technique

All surgeries were performed by two attending plastic surgeons (D.Y.O. and J.H.L.) in identical fashion. A reliable perforator was preoperatively selected based on the size and length of the intramuscular segment and the connection with superficial venous system on multidirectional CT. Two perforators with a single row were generally included in a flap. However, it could be changed based on the intraoperative findings. The surgeons intraoperatively skeletonized all selected perforators until the fascial slit was visualized using suprafascial dissection (Video supplement 1). Facial incision was performed through the slit around the perforator, and subfascial dissection of perforator was performed to free any attachment between the perforator and rectus fascia. When multiple perforators need to be selected, we have tried to select them from the same row of pedicle. In addition, we performed fascial incision in a zigzag fashion when the perforators were horizontally apart. This was followed by an intramuscular dissection of the pedicle in a usual manner. Motor nerves to the rectus muscle were saved unless the perforator harvest needed nerve division. Microsurgical nerve coaptation was not performed when divided. After flap harvest, the muscle and fascial layers were primarily closed layer by layer. Fascial reinforcement inlay or onlay graft was not used in all patients. Two suction drains were placed in the subcutaneous space, and they were removed if <30 cc per day over two consecutive days. Postoperatively, compressive abdominal support bandage was applied for 1 month, except when sleeping.

## 4. Statistical Analysis

The normal distribution for the continuous variable was verified using the Kolmogorov-Smirnov test. We performed the Mann–Whitney test according to the results of the normality test of the continuous variable. In contrast, we conducted Fisher's exact test for the categorical variables. In the correlation analysis, the whole set was classified according to the dependent variables. This analysis confirmed the correlation within each group. We verified the variables for regression analysis through univariate analysis. We derived the significant variables and performed multivariate analyses. A logistic regression analysis determined the risk factors for complications and odds ratio. A *p* value < 0.05 was considered statistically significant.

## 5. Results

Table 1 summarizes the overall characteristics of the patients. The mean age of 461 patients was 48.7 years, ranging from 25 to 70 years. The mean follow-up period was 22.7 months. While 10 patients (2.2%) had a history of smoking, 451 patients (97.8%) had never smoked. In addition, 29 patients (6.3%) had diabetes. The mean BMI was 24.1 kg/m^2^. The range of BMI was 17.8 to 42.5 kg/m^2^. The number of normal weight (BMI < 25), overweight (BMI 25-30), and obese (BMI > 30) patients were 302, 133, and 26, respectively. Moreover, 192 patients (41.6%) had a history of abdominal operation. Pfannenstiel incision, including cesarean delivery, was most common (*n* = 115). A total of 108 patients (23.4%) received neoadjuvant chemotherapy.

Table 2 outlines the intraoperative details. The mean weight and height of the harvested abdominal flaps were 889.7 g and 13.8 cm, respectively. We measured the length from the xiphoid process to the symphysis pubis on preoperative CT scan. We assumed that the relative flap height ratio was the flap height divided by the length from the xiphoid process to the symphysis pubis. The mean value of the ratio was 0.395. Unilateral dissection was performed in 299 patients (64.9%), compared to bilateral dissection in 162 patients (35.1%). All cases of TRAM flap dissection were elevated as a muscle sparing type II technique.

There were 33 patients (7.2%) with donor site complications (Table 3). Of these patients, two had abdominal bulging. However, there was no case of abdominal hernia. There were two and four cases of abdominal hematoma and seroma, respectively. In addition, delayed wound healing occurred in 28 patients. One patient had both bulging and delayed wound healing.

Table 4 shows the risk factors associated with the complications. The univariate analysis did not reveal a significant association between the preoperative characteristics and the development of complications. The DIEP group showed a higher complication rate (7.5%) than the TRAM group (4.3%), but there was no significant difference (*p* = 0.761). The complication rate was higher in the bilateral dissection group (10.5%) than in the unilateral group (5.4%), but it was not significant (*p* = 0.057). Despite the significantly higher flap height in the complication group (14.4 cm vs. 13.8 cm, *p* = 0.026), there was no substantial difference in the flap weight between the groups. Chemotherapy and the relative flap ratio did not significantly differ between the two groups.

Table 5 summarizes the variables that had a significant influence on the individual complications. The history of smoking was significantly associated with the development of abdominal bulging (*p* = 0.043). BMI was substantially correlated with hematoma (*p* = 0.024). Moreover, delayed wound healing was significantly associated with the height and weight of flap (*p* = 0.002 and *p* = 0.010).

We performed logistic regression analysis with the significant variables shown in Table 5. BMI, a significant variable for hematoma, was not statistically significant on the logistic regression analysis (Table 6). The flap height was found significant on performing a multivariable logistic regression analysis with the flap weight and height as significant variables of delayed wound healing. Moreover, the risk of delayed wound healing increased 1.396 times with an increase in the flap height. Smoking experience was analyzed as a factor that had a significant influence on bulging, but because there were only 2 cases of bulging, it would be difficult to find statistical significance, so regression analysis was not performed.  Table 7 summarizes the details of the two patients with bulging. They were aged 57 and 59 years. This was slightly greater than the overall average age of 48 years. Both patients received bilateral dissection of pedicle for unilateral reconstruction. In addition, they received adjuvant chemotherapy after surgery and had a history of abdominal operation. In addition, the bulging appeared 9 months postoperatively in both patients. One patient was overweight (*BMI* = 29) and had diabetes and a history of smoking.

## 6. Discussion

Both the rectus muscle and the anterior rectus sheath can get damaged and resected during flap elevation. Thus, dissection techniques were developed to preserve the rectus muscle to the maximum extent possible, which finally evolved to the DIEP flap. This in turn helped decrease the donor site morbidity, such as abdominal bulging and hernia.

However, the reported incidence of hernia or abdominal bulging is different within the same type of flaps (traditional TRAM vs muscle sparing TRAM vs DIEP) [6–8]. In addition, Nahabedian et al. showed that there is no significant difference in the rate of lower abdominal bulging between muscle sparing and nonmuscle sparing techniques, except for the bilateral free TRAM flap [9]. This can be attributed to the classification of the abdominal flap that is predominantly focused on the amount of muscle sacrificed during the flap elevation. However, according to prior studies, the amount of the resected anterior rectus sheath, not the amount of the resected rectus muscle, most likely predisposes to abdominal bulging [10–12]. Erni and Harder [12] reported on no incidence of bulging or hernia in 20 patients of free or pedicled fascia-preserving TRAM flap. Heo et al. reported on the fascia being spared and primarily closed in 5.4% cases of donor site morbidity and 1.3% cases of abdominal hernia in 615 patients with type 1 muscle-sparing free TRAM flap [13]. This in turn is comparable to the reported incidence of hernia in the DIEP flap.

Some studies have recommended reinforcing the anterior rectus sheath with various onlay or inlay graft to decrease donor site complication [4, 14]. However, the indication of use is unclear. Furthermore, researchers have proposed different indications. For example, Leon et al. considered using mesh according to the condition of fascia, patient obesity, extent of muscular dissection, and bilateral or unilateral harvesting [8]. In addition, another study considered bilateral and obese patients to have higher rates of hernia and bulging. Thus, prophylactic mesh or cadaver dermis was used in the patients [7]. Apart from the absence of a clear indication, its use was dependent on the “operating surgeon's decision” [4, 8, 15]. Even when the fascial could be closed primarily, the inlay or onlay grafts were used at the discretion of the surgeons. Nonetheless, graft materials have their disadvantages, such as infection and delayed wound healing. Hence, there should be a clearer indication for the use of inlay graft. Thus, we hypothesized that a graft might be unnecessary when the fascia is truly preserved.

Nahas et al. defined the traction index as the force required to pull the anterior rectus sheath towards the midline after a fascial incision [16]. The resection and closure of a portion of the rectus sheath can increase intra-abdominal pressure and traction index, which result in abdominal bulging or hernia. To decrease the traction index postoperatively, we took efforts to skeletonize the perforators until the fascial slit was visible. Through the slit, we performed fascial incision and preserved the anterior rectus sheath to the maximum extent possible. Less than 5 mm of the rectus sheath is included in the flap, when the slit is invisible or the sheath is adherent to perforators.

**Figure 1:**
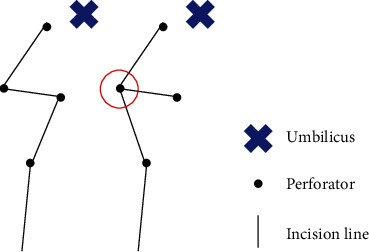
Diagram of fascial incision. Red circle refers to “weak point” after fascial closure.

The method of fascial incision is also important for maintaining its integrity. We prefer incorporating two or three perforators in the flap, following which the perforators in the same row (medial or lateral) are selected. However, the selected perforators are sometimes located apart in different rows. In this situation, we incised the fascia in a zigzag fashion and avoided a horizontal extension line. The point where the two lines meet acts as a weak point when the incision line is a combination of longitudinal and horizontal line (Figure 1).

In addition, we applied an external abdominal bandage to support the lower abdomen for 4 weeks postoperatively. According to a study that evaluated the durability of the anterior rectus sheath, one could measure the radiographic evidence of fascial separation after abdominoplasty, which did not progress after 3 weeks [17]. Hence, an application of the external abdominal bandage can decrease the tension over the suture site during the critical healing period of the fascia.

Bilateral harvesting of flap was considered one of significant risk factors for developing hernia or bulging [7]. This is important for Asian patients because they have less redundant abdominal soft tissue compared to their Western counterparts and this calls for the need of bipedicled abdominal flaps to prevent postoperative fat necrosis. In our study, bilateral dissection of pedicle was performed in 162 (35.1%) patients, but it was not significantly associated with the development of complications. There was no case of postoperative hernia in the bilateral pedicle dissection group, and bulging developed in 2 of the 162 patients. This study indicates that according to the undermentioned principle of the fascial sparing technique, donor site morbidity can be minimized even without reinforcement graft during bilateral dissection.

Besides hernia and bulging, we evaluated the other donor site morbidities and their risk factors. The risk of overall complication was significantly associated with the flap height and it was similar to that obtained previously [18]. An increase in the height flap by one was concomitant with an increase of the risk of overall complication by 1.299. We also investigated the association between the relative height of the flap and the development of abdominal complications. However, we found an association between the vertical height per se and the development of complication. Moreover, the relative height was not significantly associated with it. Though we expected that a shorter abdominal length remaining after flap harvesting would affect the incidence of complications, the results were not so. This may be because of the simplicity of length measurement on CT, which does not take the elasticity of the tissue into consideration. That is, even when the length of the abdominal tissue remaining after harvesting the flap is short (i.e., the relative height of the flap is long), there may be no significant difference from the group with a short flap height if it can be stretched well during suture and the tension of the closure site decreases. While abdominal bulging was associated with smoking, hematoma was associated with BMI. In addition, the risk of delayed wound healing increased with an increase in the flap weight and height. An increase in the height flap by one augmented the risk of delayed wound healing by 1.396.

The retrospective design of our study was a major limitation. Furthermore, the risk factor for abdominal bulging was less reliable because of its low incidence. In addition, the BMI of our patients was relatively low compared to that of Caucasians. Abdominal complications in patients with a BMI < 30 is less than those in patients with a higher BMI [5]. Majority (94.3%) of our patients had BMI < 30. Thus, further study for obese patients is warranted.

## 7. Conclusions

Breast reconstruction with an abdominal free flap can be safely performed with a low incidence of abdominal bulging. This study showed that there was no significant difference in the incidence of complications even in the bilateral dissection of the pedicle without fascia reinforcement. The fascia should be preserved to minimize postoperative abdominal bulging or hernia. In addition, a zigzag fascial incision is preferred during the incorporation of multiple perforators. This will minimize abdominal complications without using inlay or onlay grafting material.

## Figures and Tables

**Table 1:** Patient characteristics (*n* = 461).

Characteristic	Value
Age at surgery (yr)	48.7 ± 7.9 (25-70)
Ever smoker	
Yes	10 (2.2)
No	451 (97.8)
Diabetes	
Yes	29 (6.3)
No	432 (93.7)
BMI (kg/m^2^)	24.1 ± 3.5 (17.8-42.5)
<25	302
25-30	133
>30	26
Previous abdominal operation history	
Yes	192 (41.6)
No	269 (58.4)
Midline	14
Pfannenstiel	115
Appendectomy	23
Laparoscopy	52
Liposuction	2
Neoadjuvant chemo	
Yes	108 (23.4)
No	353 (76.6)

Values are presented as mean ± standard deviation (range) or number (%).

**Table 2:** Operative details.

Variables	Value
Flap weight (g)	889.7 ± 380.9 (250-2836)
Flap vertical height (cm)	13.8 ± 1.4
Flap height/trunk ratio	0.39545 ± 0.042
Dissection of pedicle	
Unilateral	299 (64.9)
DIEP	261
TRAM	38
Bilateral	162 (35.1)
Bilateral DIEP	154
Bilateral TRAM	2
DIEP+TRAM	6

Values are presented as mean ± standard deviation (range), mean ± standard deviation, or number (%). DIEP: deep inferior epigastric artery perforator flap; TRAM: transverse rectus abdominis musculocutaneous flap.

**Table 3:** Postoperative complications.

	Value
Hernia, *n* (%)	0 (0.0)
Bulging, *n* (%)	2 (0.4)
Hematoma/seroma, *n* (%)	4 (0.9)
Delayed wound healing, *n* (%)	28 (6.1)
Total, *n* (%)	33 (7.2)

**Table 4:** The impact of patient and surgical variables on complications.

	Overall donor-site complications		*p* values
	No (*n* = 428)	Yes (*n* = 33)	
Mean age at surgery (yr)	48.5	50.5	0.106
Ever smoker			0.156
Yes	8 (80)	2 (20)	
No	420 (93.1)	31 (6.9)	
Diabetes			1
Yes	27 (93.1)	2 (6.9)	
No	401 (92.8)	31 (7.2)	
Mean BMI (kg/m^2^)	24.1	24.9	0.703
Previous abdominal operation history			0.856
Yes	179 (93.2)	13 (6.8)	
No	249 (92.6)	20 (7.4)	
Dissection of pedicle			0.057
Unilateral	283 (94.6)	16 (5.4)	
Bilateral	145 (89.5)	17 (10.5)	
Mean flap weight (g)	880.6	1010.9	0.07
Type of flap			0.761
DIEP, *n* (%)	384 (92.5)	31 (7.5)	
TRAM, *n* (%)	44 (95.7)	2 (4.3)	
Neoadjuvant chemotherapy			0.835
Yes	101 (93.5)	7 (6.5)	
No	327 (92.6)	26 (7.4)	
Flap height (cm)	13.8	14.4	0.026^∗^
Flap height/trunk ratio	0.394	0.408	0.363

Values are presented as number (%). DIEP: deep inferior epigastric artery perforator flap; TRAM: transverse rectus abdominis musculocutaneous flap. ^∗^Statistically significant.

**Table 5:** Variables significantly associated with individual complication.

Complication	Variables (*p* value)
Bulging	Smoking experience (0.043)
Hematoma	BMI (0.024)
Delayed wound healing	Flap weight (0.010)
	Flap height (0.002)

**Table 6:** Logistic regression on total complication.

	OR (95% CI)	*p* value
Logistic analysis on hematoma		
BMI	0.589 (0.343-1.011)	0.055
Logistic regression on delayed wound healing		
Flap weight	1.000 (0.999-1.001)	0.882
Flap height	1.396 (1.007-1.935)	0.046

BMI: body mass index.

**Table 7:** Details of the patients with bulging.

Pt.	Complication	Age (yr)	BMI (kg/m^2^)	Smoking	P/Hx	Bipedicled flap	Flap weight (kg)	Adjuvant CTx.	Previous abd op	Time to bulging after op	Flap
1	Bulging	59	18.99	None	None	Yes	458	(+)	Laparoscopy	9m14d	Bipedicled DIEP
2	Bulging wound dehiscence	57	29.06	Exsmoker	DM	Yes	1120	(+)	Pfannenstiel	9m24d	DIEP & ms-2 TRAM

## Data Availability

The data used to support the findings of this study are available from the corresponding author upon request.

## Supplementary Materials

Supplementary Materials Skeletonization of perforator and complete preservation of the fascia.
